# Genomic characterization of *canine circovirus* associated with fatal disease in dogs in South America

**DOI:** 10.1371/journal.pone.0218735

**Published:** 2019-06-25

**Authors:** Fiorella Kotsias, Danilo Bucafusco, Denise Anabel Nuñez, Lucía Antonella Lago Borisovsky, Mariana Rodriguez, Ana Cristina Bratanich

**Affiliations:** 1 Cátedra de Virología, Facultad de Ciencias Veterinarias, Universidad de Buenos Aires (UBA), Buenos Aires, Argentina; 2 Instituto de Investigaciones en Producción Animal (INPA), Consejo Nacional de Investigaciones Científicas y Técnicas (CONICET) – Universidad de Buenos Aires (UBA), Buenos Aires, Argentina; 3 Consejo Nacional de Investigaciones Científicas y Técnicas (CONICET), Argentina; 4 DVM, Private practice, Buenos Aires, Argentina; Cornell University, UNITED STATES

## Abstract

*Canine circovirus* (CanineCV) was detected, together with *canine parvovirus* (CPV), in samples from an outbreak of fatal gastroenteritis in dogs in Argentina. We obtained the full-length genome of this recently discovered virus by overlapping PCR, designated strain UBA-Baires. Sequence analysis revealed a highly conserved genome but also showed several unique mutations in amino acids from the capsid protein that have not been previously reported. Phylogenetic analysis shows that this strain is more closely related to European strains than to viruses detected in North America or Asia. Although the pathogenic role of CanineCV in dogs is still unclear, this study highlights the importance of CanineCV as a coinfecting virus in disease development. To our knowledge, this is the first report of the involvement of CanineCV in severe clinical disease in dogs in South America. Our results expand our information on the geographical extent of this virus and contribute to the understanding of its role in disease.

## Introduction

A newly described *canine circovirus* (CanineCV) was identified recently in serum from healthy dogs [1]. Since then, several countries have reported the molecular detection of CanineCV in cases associated with clinical disease, ranging from sudden death of puppies with bloody diarrhea to respiratory syndromes [2–6]. Lesions found include necrotizing vasculitis, lymphoid necrosis, granulomatous inflammation and necrosis of intestinal crypts and Peyer’s patches. Some characteristics of the disease observed in dogs (enteritis, pneumonia, lymphadenitis) were also present in pigs infected with *porcine circovirus 2* (PCV-2), which was found to be genetically related [7].

A number of case-control studies have been published, most of them describing significant association between diarrhea and CanineCV detection [8–11]. The role of this new virus in pathogenesis is however difficult to assess since it is also found in feces from healthy dogs. Furthermore, the first description was done from serum samples from six healthy animals [1] and there have been recent reports of CanineCV in serum samples of dogs from Brazil and China [12, 13]. Additionally, coinfection with other viral pathogens, mainly *canine parvovirus* (CPV), but also *canine distemper virus*, *canine coronavirus*, and *canine influenza virus* is present in CanineCV positive dogs with clinical symptoms at rates between 50 to 100% [4, 6, 8–11]. Viral coinfection is also a main feature of PCV-2 infection [14, 15].

Circoviruses belong to the family *Circoviridae*, genus *circovirus*; they are small, non-enveloped virus with a circular single strand DNA genome of around 2000 bp [16]. The genome contains two main (and opposite) transcription units which encode two ORFs, a replicase associated protein (Rep) and the capsid protein (Cap). CanineCV sequences submitted to Genbank are quite similar differing slightly from those identified in wildlife. Nevertheless, the division of CanineCV into two genotypes (CanineCV-1 and Canine CV-2) based on the *cap* gene sequence has been proposed very recently [12].

In this work, we present, to our knowledge, the first report of a complete full-length CanineCV genome sequence from South America.

## Materials and methods

### Ethics statement

This study did not involve any animal experiments. Tissue samples were collected during necropsy of dead animals for laboratory analysis, with consent from the dog’s owner.

### Animal description and sample collection

Severe disease outbreaks occurred in a mixed-breed colony in Buenos Aires between 2014 and 2015, in a cascade fashion. The age of affected dogs ranged from 50 days to one year, belonging to different breeds. The attending veterinarian described the situation as a highly contagious disease of very rapid onset and development, characterized by anorexia, vomiting and hemorrhagic diarrhea lasting from three to 7 days. In total, 18 puppies died, and the rest recovered within a week. All dogs had been properly vaccinated against CPV, canine distemper virus and infectious canine hepatitis virus. Fresh tissues obtained from pancreas, liver, kidney, lung, lymph nodes, spleen, large intestine and stomach belonging to three dead animals were submitted to the Virology Department of the Faculty of Veterinary Sciences at the University of Buenos Aires for testing. Samples belonging to surviving animals or to the other dead dogs were not available.

### DNA extraction and PCR detection

The presence of CanineCV DNA was analyzed by PCR. Tissues were processed for DNA extraction using DNazol (Invitrogen). One hundred ng of DNA were used in a PCR reaction using 25 pmoles of each primer set (Table 1), 1X reaction buffer, 1 unit GoTaq (Promega), 200 μM dNTPs in a total volume of 50ul. Primers were designed according to conserved sequences of CanineCV deposited in Genbank using the Primer-BLAST program tool (NCBI-NIH). In a first approach, the primer set *For genomic/Rev 533* was utilized to amplify a 533 bp fragment from the Rep gene for diagnostic purposes, after which positive samples were further tested with other primer sets in order to cover the entire genome for sequencing purposes (Table 1).

**Table 1:** List of primers used in this study.

Primer sequence 5’-3’	Name and location	Genomic position
ATGGCTCAAGCTCAGGTTG	*For genomic*	1–20
CTTGCGCGAGCTGCTCCTTA	*For 456*	456–478
CCGCACAGAACCTCCACTTC	*Rev 533*	514–533
AAT AAACAGCCT CGAAGGGG	*Rev 1055*	1036–1055
GTTCCATAGCAACGGGGTCT	*For 1734*	1734–1753
CATGCGCGTGCGGAGACATG	*Rev 1910*	1910–1929
GTCGGAGGAGCGGAGGTGT	*Rev genomic*	2045–2063

Samples were additionally tested for CPV. Two μL (10 ng) of extracted DNA were used in a PCR reaction that amplifies a 583 bp fragment of the VP2 gene using 555*for* and 555*rev* primers; this fragment includes a MboII restriction site identified by Buonavoglia et al. which allows the differentiation of CPV-2c strains from other CPV strains [17]. The resulting amplicon was digested with MboII to detect the presence of the CPV-2c strain´s characteristic substitution in VP2 gene (T to A at nt 4026) as described previously [17].

### Full-genome sequencing of CanineCV strain

In order to determine the full-length genome of the CanineCV strain from Argentina, a positive sample was tested with the additional primer sets listed in Table 1 and sequenced twice. Resulting amplicons were eluted after agarose gel electrophoresis with Easy Pure Quick gel Extraction kit (TransGen Biotech) and submitted twice for sequencing to Unidad Genómica, Instituto de Biotecnología (INTA, Argentina) and to GenBiotech (Buenos Aires, Argentina), following the dideoxy-nucleotide chain termination method using the primers described above. The full-length genome was assembled using the Mafft software (EMBL-EBI). The complete sequence was named strain UBA-Baires and deposited in Genbank under accession number MK033608.

### Sequence analysis

For phylogenetic analysis of the complete CanineCV genome sequence, we set up a matrix that included the 22 different complete CanineCV sequences deposited so far in GenBank. Details of selected sequences are shown in Table 2. This data set was organized using BioEdit software (v. 7.2.5) [18] and aligned with ClustalW (version 1.82)[19]. A maximum likelihood (ML) phylogenetic tree of these sequences was obtained using PhyML (version 3.1) [20]. For this analysis, we utilized the TN93+I+G model of nucleotide substitution (determined using Smart Model Selection) [21, 22] with sub-tree pruning and regrafting (SPR) tree improvement and 10 starting random trees. The robustness of individual nodes on the phylogeny was estimated using 1000 bootstrap replicates. Phylogenetic trees were visualized and edited with FigTree v1.4.3 (http://tree.bio.ed.ac.uk/software/figtree/).

**Table 2:** Full-length genome sequences of CanineCV selected for phylogenetic analysis.

GenBank Accession Number	Origin	Year	Host	Virus ID	Reference
MK033608	Argentina	2018	dog	UBA-Baires/2018/ARG	This study
JQ821392	USA	2012	dog	214/2012/USA	Kapoor et al., 2012
KC241982	USA	2013	dog	UCD1/2013/USA	Li et al., 2013
KC241983	USA	2013	dog	UCD3/2013/USA	Li et al., 2013
KC241984	USA	2013	dog	UCD2/2013/USA	Li et al., 2013
KF887949	Germany	2014	dog	Ha13/2014/GER	Halami et al., 2014 (unpublished)
KJ530972	Italy	2014	dog	Bari/2014/ITA	Decaro et al., 2014
KT283604	Germany	2015	dog	FUBerlin/2015/GER	Plog et al., 2015 (unpublished)
KT946839	China	2015	dog	JZ98/2015/CHI	Zheng et al., 2015 (unpublished)
KT734812	Italy	2016	wolf	CB6293/2016/ITA	Zaccaria et al., 2016
KT734813	Italy	2016	dog	AZ2972/2016/ITA	Zaccaria et al., 2016
KT734814	Italy	2016	wolf	TE4016/2016/ITA	Zaccaria et al., 2016
KT734815	Italy	2016	wolf	AZ4133/2016/ITA	Zaccaria et al., 2016
KT734816	Italy	2016	badger	AZ4438/2016/ITA	Zaccaria et al., 2016
KT734817	Italy	2016	dog	AZ5212/2016/ITA	Zaccaria et al., 2016
KT734819	Italy	2016	wolf	AZ5586/2016/ITA	Zaccaria et al., 2016
KT734820	Italy	2016	wolf	AZ663/2016/ITA	Zaccaria et al., 2016
KT734821	Italy	2016	dog	TE6685/2016/ITA	Zaccaria et al., 2016
KT734822	Italy	2016	wolf	TE7482/2016/ITA	Zaccaria et al., 2016
KT734823	Italy	2016	dog	PE8575/2016/ITA	Zaccaria et al., 2016
MG266899	China	2017	dog	CD17/2017/CHI	Yan et al., 2017 (unpublished)
MF797786	China	2018	dog	XF16/2018/CHI	Je et al., 2018 (unpublished)
MF457592	USA	2018	dog	OH19098/2018/USA	Wang et al., 2018 (unpublished)

## Results

### Detection of CanineCV

**Fig 1:**
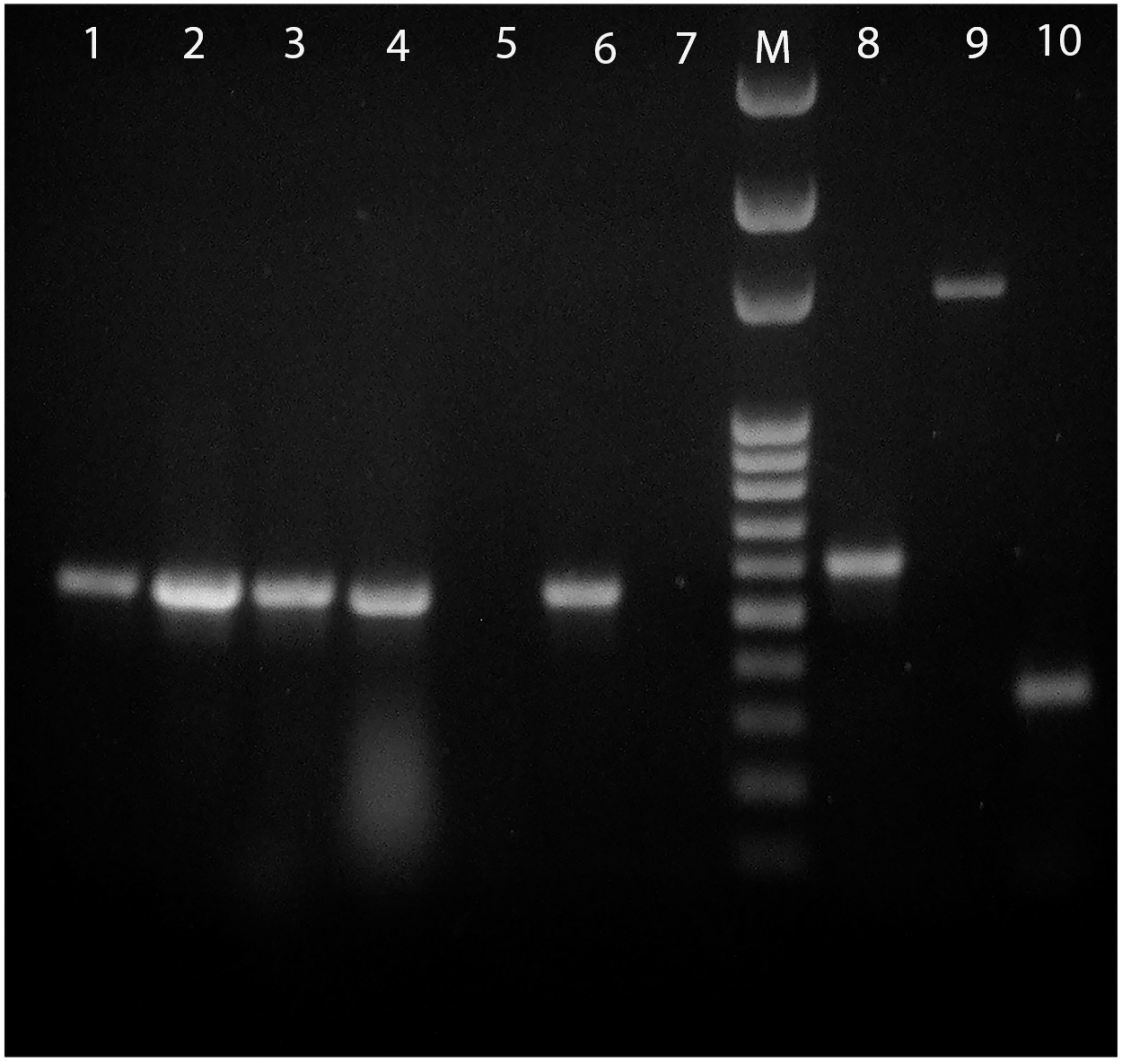
Detection of CanineCV. Gel electrophoresis of PCR reactions for CanineCV with different tissues and primers. Lanes 1–7 set *For genomic*/*Rev 533*. *Lane 1*: spleen, 2: mesenteric lymph node, 3: liver/lung, 4: pancreas, 5: stomach, 6: large intestine, 7: negative control. Line 8: *For 456/Rev 1055*, Line 9: *For 456/Rev 1910*, Line 10: *For 1734/Rev genomic*. M: molecular weight marker 100 bp DNA Ladder (Solis BioDyne).

**Fig 2:**
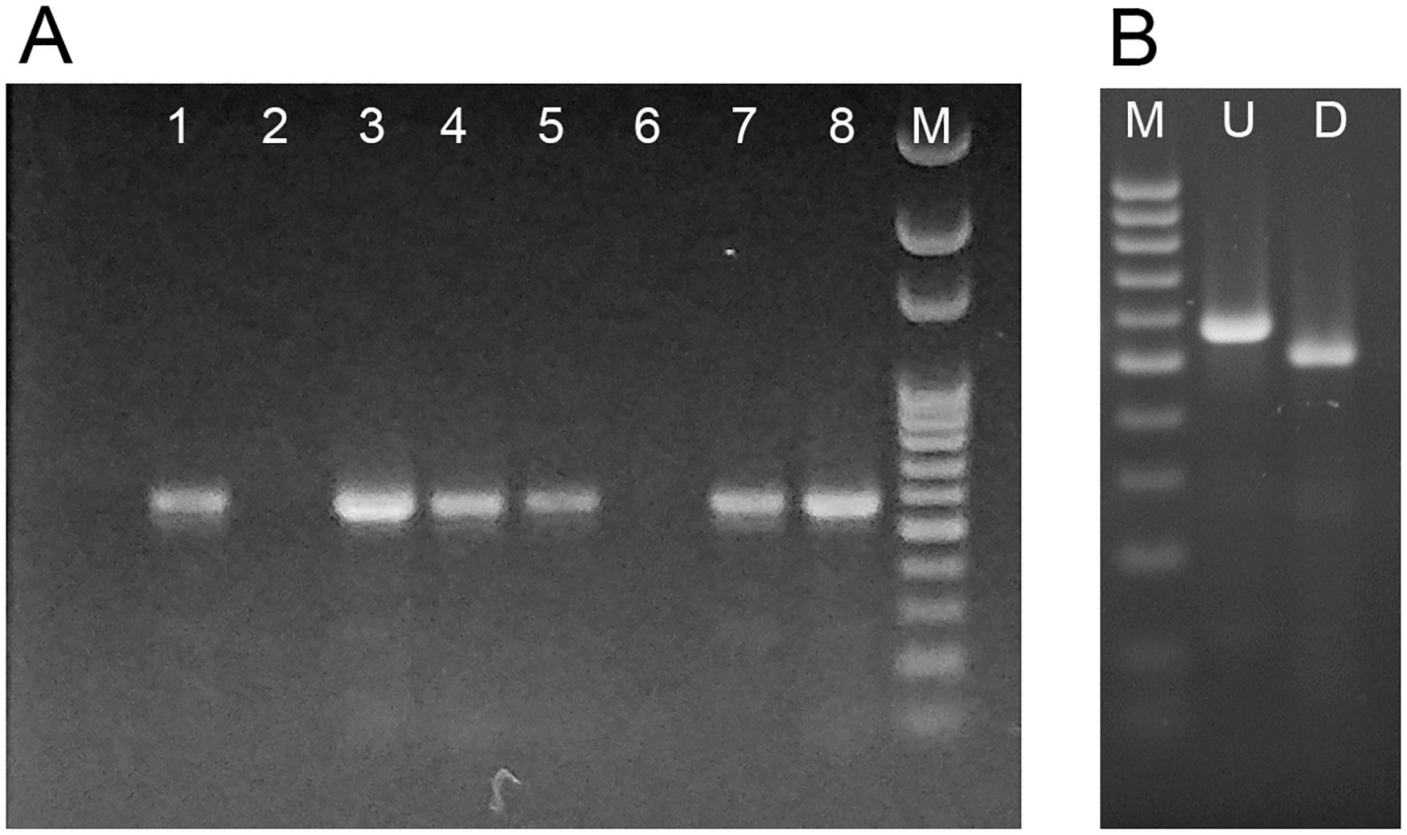
Detection of CPV. Gel electrophoresis of (A) PCR reactions for CPV. Lane 1: positive control, 2: negative control, 3: Mesenteric lymph node, 4: Spleen, 5: Lung, 6: Stomach, 7: Liver, 8: Large intestine, M: Molecular weight marker 100 bp DNA Ladder (Solis BioDyne). (B) MboII Restriction Fragment Length Polymorphism of Large intestine amplicon. M: Molecular weight marker 100 bp Ladder, U: Undigested Large intestine amplicon, D: MboII digested Large intestine amplicon.

Pooled tissue samples from two of the three animals tested positive for CanineCV with the primer set *For genomic/Rev 533*. Then, individual organs were further screened with the same primer set finding that all tissues (pancreas, liver, kidney, lung, lymph nodes, spleen and large intestine), except for the stomach, were positive as well. DNA from one of these tissues (lymph node) was randomly selected for further analysis with the remaining primer sets in order to assemble the entire CanineCV genome sequence, which was concluded to be of 2063 bp (Fig 1). PCR analysis detected the presence of CPV in all samples, except for the stomach (Fig 2A). Moreover, all generated amplicons were digested with MboII, indicating the presence of the glutamine at VP2 aa 426 characteristic of CPV-2c strains [17] (Fig 2B).

### Genome characterization

Sequence analysis revealed strong similarities to those of other previously described CanineCV. The complete genome of strain UBA-Baires was 2063 nt in length with a GC content of 51.5%. Genome characteristics include two putative ORFs synthesized from both DNA strands and two intergenic regions. These ORFs encode a putative viral replicase (Rep) and a capsid protein (Cap), which are 303 and 270 aa long, respectively. The 5’ intergenic region, located between the start codons and encompassing 135 nt, contains a conserved stem-loop structure and a 9 nt sequence (TAGTATTAC) for initiation of rolling cycle replication. The overall nt identity of strain UBA-Baires ranged from 98% (strain Bari/411-13) to 85% (isolate UCD3-478). When compared to other CanineCV, both Rep and Cap ORFs were, as expected, highly conserved including sequences related to rolling circle replication (FTINN, TPHLQ and CSK) and dNTP binding (GCGKS) in the non-structural gene (*Rep*). However, four aa changes within the *Cap* gene and one within the Rep gene were found, which have not been previously reported for other CanineCV sequences being unique to strain UBA-Baires (Table 3). These mutations were confirmed after two independent sequencing runs directly from PCRs amplicons. No mutations were observed in the intergenic regions.

**Table 3:** Amino acid substitutions in the replicase and capsid proteins unique to CanineCV strain UBA-Baires.

Protein name	Nt position	Nt substitution	Protein residue position	strain UBA-Baires	CanineCV strains
Replicase	580	C to G/A	194	Gln	Glu / Lys
Capsid	1499	T to A/G	144	Cys	Ser / Gly
	1522–1523	CA to AC	136	Gln	Thr
	1645	T to A	95	Phe	Tyr
	1694	A to G	79	Thr	Ala

### Phylogeny

**Fig 3:**
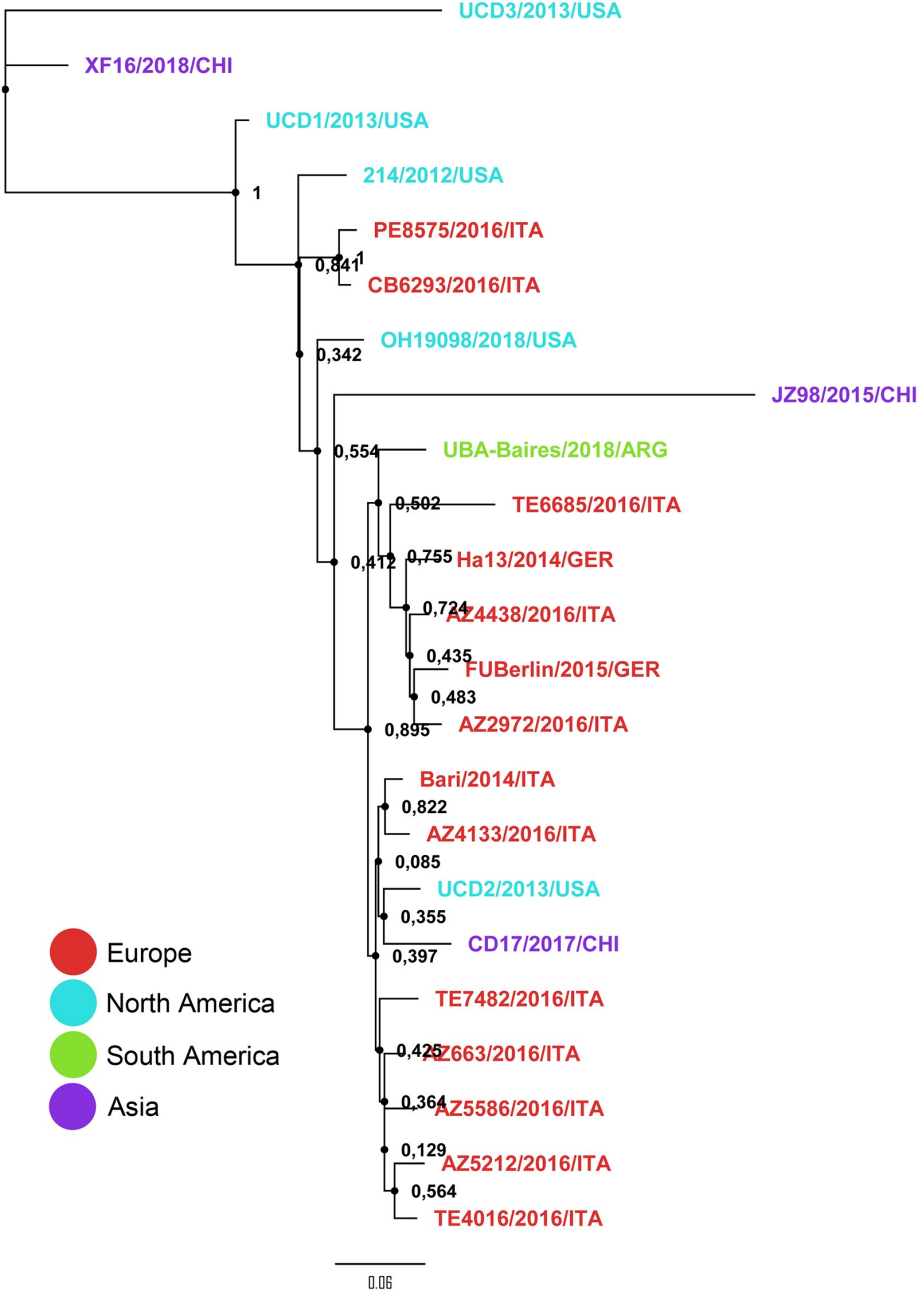
Maximum likelihood tree of full-length sequences of CanineCV strains. The sequences are colored based on their continental origin. Details of data set are shown in Table 2.

Phylogenetic evaluation of the genetic relationship with other CanineCV was performed using whole genome sequences published so far (Table 2). Among the CanineCV sequences, the strain from the present study clustered with viruses circulating recently in animals from Europe, mainly Germany and Italy, with strong bootstrap support, and was divergent from most strains from the USA and China (Fig 3).

## Discussion

Kapoor et al. reported for the first time the detection of CanineCV in the serum of healthy dogs in the USA [1]. Over the last five years, CanineCV has been detected in Italy, Germany, China, Thailand, Taiwan and Brazil [3–6, 8, 12, 13]. In the present study, we report an outbreak of a fatal disease associated with the presence of CanineCV in Argentina.

Although virus isolation was not attempted since it was proved unsuccessful in previous reports [3], the full-length genome of strain UBA-Baires was determined, revealing close similarity to European viruses. We found only one unique mutation in the *rep* gene, but surprisingly, four unique aa substitutions in the Cap protein, after two independent experiments using different sequencing facilities. Very recently, nine major variable regions have been proposed to exist within the *cap* gene of CanineCV [12]. Only one of the four mutations present in the *cap* gene of strain UBA-Baires is encompassed at these proposed locations (residue 95), suggesting that, until sequences from more extent geographical areas are analyzed, these variable regions need to be taken cautiously. Point mutations in the *cap* gene have been described for PCV2 and are a potential source of virus evolution, especially due to selective pressure caused by vaccination [23]. In the case of CanineCV, where vaccination has not been used, the mechanisms behind these aa substitutions are unclear; virus variability under natural infection has been shown to be very complex for PCV-2 [24]. In addition, the relevance of these changes, if any, is difficult to be evaluated at this moment when it is still under investigation the role of CanineCV as a sole cause of disease [25].

In the case of PCV2, coinfection with other pathogens has been associated with different clinical outcomes [14]. Special attention was given to coinfection with porcine parvovirus and the syndrome could be reproduced experimentally [26, 27]. Similarly, CanineCV has been found in most cases accompanying other viral pathogens. In hemorrhagic enteritis, it is often associated to CPV, but also canine distemper virus and canine coronavirus with coinfection rates of 100% [4, 6], 70–80% [8, 9, 11] and 50% [10]. Only one report so far has found CanineCV as a primary agent [3]. Case-control studies carried out in different population of dogs suggest a high association between diarrhea and CanineCV, but CanineCV is still found in feces and serum from healthy dogs [8–11]. Nevertheless, one study showed that mortality rate was higher when CanineCV was found together with CPV [8]. Interestingly, in dogs with respiratory symptoms from Thailand, CanineCV was detected together with respiratory pathogens (*canine influenza virus*, *canine parainfluenza virus*, and *canine respiratory coronavirus*)[5]. In our case, all organs tested except for stomach, were also positive for CPV. Unfortunately, no histopathology was performed on these cases in order to confirm the presence of some of the lesions observed in previously described cases involving CanineCV, in particular vasculitis [25]. Surprisingly, in our case, sick animals had been vaccinated against CPV. This phenomenon was also described in a breeding colony in the USA where two severe outbreaks of hemorrhagic gastroenteritis occurred among animals vaccinated against CPV [4]. Again, the role of CanineCV in the context of vaccine efficacy against co-infecting viruses is still unclear and needs to be carefully analyzed. ISH studies carried out with clinical samples show that CanineCV infects mainly lymphoid tissues and cells [2, 5] suggesting that Infection with circovirus could cause immunosuppression and allow infection with CPV even in the case of vaccinated animals. Nevertheless, further experimental infections are necessary to elucidate the pathogenic involvement of CanineCV in disease pathogenesis.

To our knowledge, this is the first report of a complete genome sequence of CanineCV associated with disease from Latin America. A very recent paper published by Weber and col. characterizing the dog’s virome found CanineCV in the sera from seven healthy dogs from Brazil [13]. Phylogenetic analysis using a partial sequence from Rep revealed that the Brazilian virus seemed to be related to strains from China and the USA, among others. The tree topology is somehow different form the one we present in this study based on the full-length genome, in which the Argentinian strain grouped closer to European viruses. Hopefully, additional sequences from South America will be soon deposited in GenBank allowing a more thorough analysis of the epidemiology and evolution of this emerging virus and will aid in the comprehension of its role in disease.
